# Averaged Impedance Drop Estimates Conduction Gap During Pulmonary Vein Isolation in Atrial Fibrillation

**DOI:** 10.1111/jce.70246

**Published:** 2026-01-09

**Authors:** Masahide Harada, Yuji Motoike, Yoshihiro Nomura, Asuka Nishimura, Eiichi Watanabe, Yukio Ozaki, Hideo Izawa

**Affiliations:** ^1^ Department of Cardiology Fujita Health University Toyoake Aichi Japan; ^2^ Department of Cardiology Fujita Health University Bantane Hospital Nagoya Aichi Japan; ^3^ Department of Cardiology Fujita Health University Okazaki Medical Center Okazaki Aichi Japan

**Keywords:** atrial fibrillation, impedance drop, pulmonary vein isolation, radiofrequency catheter ablation

## Abstract

**Background:**

EnSite X provides a module to filter oscillated generator impedance signals due to cardiac beating/respiration (averaged impedance drop, AID). TactiFlex ablation catheter Sensor Enabled (TFSE) is the latest product to incorporate a contact‐force sensor with a flexible tip but without lesion‐estimating parameters.

**Methods:**

To examine if AID estimates lesion formation, AID were compared between ablated lesions (*n *= 1687) with and without conduction gaps (CGs) after first‐pass pulmonary vein isolation (PVI) using TFSE in atrial fibrillation (AF) patients (*n* = 20). The clinical efficacy of AID‐guided PVI was evaluated in another subset of AF patients (*n* = 30).

**Results:**

CGs were observed in 45 points (CG[+]) but not in 1642 points (CG[−]). CG[+] had lower %AID (=absolute AID/initial impedance, *p* < 0.0001) than CG[−]. Decrease of bipolar voltage amplitude during radiofrequency application was linearly correlated with %AID (*R* ^2^= 0.785, *p* < 0.05). In receiver operating curve analysis, the cutoff values of %AID and unfiltered generator impedance drop for predicting CG was 9.33% and 11.0 Ω, respectively; %AID had significantly higher area under the curve than unfiltered generator impedance drop (0.761 vs. 0.627, *p* < 0.05). In swine heart experiments, %AID was correlated with lesion volume (*R* ^2^ = 0.711, *p* < 0.05). In another subset of AF patients, success rate of first‐pass isolation in %AID ( ≥ 9%)‐guided PVI was 90% for LPV and 90% for RPV. The 1‐year event‐free rate of atrial tachyarrhythmias was 82%.

**Conclusion:**

AID would improve the usability of impedance drop as an end point for RF　application. %AID‐guided PVI using TFSE would be effective in AF patients.

## 1 Introduction

**Figure 1:**
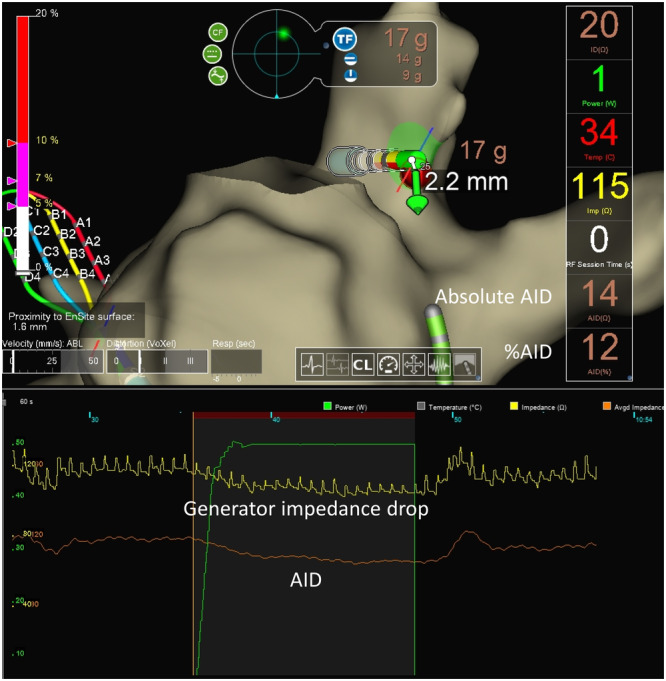
Generator impedance drop and averaged impedance drop (AID).

Generator impedance drop is utilized to estimate lesion formation in radiofrequency (RF) catheter ablation [1, 2]. However, cardiac beating and respiration usually cause oscillation of impedance signals, reducing the accurate estimation of lesion formation. The latest 3‐dimensional mapping system (EnSite X, Abbott) provides an Aid Module to eliminate the oscillation artifact by filtering original impedance signals (averaged impedance drop, AID, Figure 1), which may improve the accuracy of estimating lesion formation of pulmonary vein isolation (PVI) in atrial fibrillation (AF).

The TactiFlex Ablation Catheter, Sensor Enabled (TFSE, Abbott, St. Paul, MN, USA) is the latest radiofrequency ablation catheter incorporating fiber optics‐based contact force sensing technology with a flexible laser‐cut tip. TFSE is reportedly safer and more effective for AF ablation than previous RF catheters [3, 4]. Although recent modules of 3D‐mapping system provided lesion‐estimating indices, such as Ablation Index and Lesion Size Index, no parameters estimate lesion formation when PVI is performed using TFSE.

This study therefore examines whether AID can estimate lesion formation of PVI using TFSE.

## 2 Methods

This study consisted of two protocols. The first study evaluated the relationship between AID and lesion formation and sought the optimal AID cutoff for predicting conduction gaps (CGs) in PVI using TFSE in AF patients. The second study validated the efficacy and safety of AID‐guided PVI using TFSE, following the first study.

### 2.1 Patients

AF patients who were planned to undergo index PVI at Fujita Health University were eligible. The review board of Fujita Health University School of Medicine approved the protocol and all patients gave written informed consent (HM20‐273). The study complied with the ethical standards of the Declaration of Helsinki. Baseline demographics and clinical information were obtained, and laboratory examinations were performed before the procedure. Transthoracic echocardiography was performed before catheter ablation to assess left‐atrial diameter (LAD), left‐ventricular systolic/diastolic dimensions, and left‐ventricular ejection fraction. Cardiac computed tomography imaging reconstructed a 3‐dimensional image of the left atrial (LA)/pulmonary vein geometry.

All patients received oral anticoagulation therapy with non‐vitamin K antagonist oral anticoagulant or vitamin K antagonist (warfarin) at appropriate doses for ≥ 4 weeks prior to hospital admission. Transesophageal echocardiography was performed 1 day before catheter ablation to detect LA thrombus [5]. All patients took oral anticoagulant without interruption during the procedure and continued oral anticoagulation therapy for at least 3 months afterwards. All anti‐arrhythmic drugs were stopped five half‐lives prior to the procedure.

Exclusion criteria are patients with previously diagnosed structural heart disease, cardiac sarcoidosis, and moderate/severe valvular heart disease; patients under 18 years old and those who were pregnant; patients with creatinine clearance (calculated by Cockcroft‐Gault formula) < 30 mL/min and those on hemodialysis; patients who had LA appendage thrombus on transesophageal echocardiography before the procedure; and patients with mechanical valves.

### 2.2 Ablation Procedure

PVI was performed using RF ablation. In all patients, the PVI strategy was used for the first session of AF ablation. No additional linear ablation in the LA was performed; only cavotricuspid isthmus ablation was permitted for documented typical atrial flutter. In all patients, the procedure was performed under deep sedation with a continuous intravenous infusion of dexmedetomidine hydrochloride and propofol, and additional boluses of midazolam under supporting positive pressure ventilation [6, 7]. Esophageal temperature was monitored throughout the procedure; temperature limit was set to 41°C.

A bolus of 5000–10 000 international units of unfractionated heparin (100–150 U/kg) was administered before trans‐septal puncture to achieve activated clotting time > 300 s. Activated clotting time was measured every 20 min after the first heparin shot and additional heparin boluses were administered to maintain the value > 300 s.

A decapolar catheter was advanced into the coronary sinus via the internal jugular vein. An 8 Fr intracardiac echocardiography catheter (Viewflex, Abbott, St. Paul, MN, USA) was inserted into the right atrium via a 10 Fr short sheath in the right femoral vein; trans‐septal puncture was performed under intracardiac echocardiography and fluoroscopic guidance. An 8 Fr long sheath (Swartz, SL1; Abbott, St. Paul, MN, USA) was then advanced into the LA.

EnSite NavX (Abbott, St. Paul, MN, USA) was used to create a 3‐dimensional electro‐anatomical voltage map of the LA using multielectrode catheter (Advisor HD grid, Abbott, St. Paul, MN, USA) and to integrate the voltage map with the computed tomography imaging reconstruction of the LA. The contrast fluoroscopy image of the LA was also obtained. PVI was achieved using a focal “point‐by‐point” catheter approach, delivering radiofrequency energy to the cardiac tissue with TFSE (irrigation flow rate: 10 mL/min for < 38°C, 13 mL/min for ≥ 38°C, temperature control mode). PVI was performed using either high‐power ablation protocol (HPSD, RF power: 50 W, RF time: 10–12 s, contact force [CF]: 5–10 g) or standard‐power long‐duration ablation protocol (SPLD, RF power: 25–30 W, RF time 30 s, CF: 10–20 g). RF ablation lesion sets encircled the PV antra using electro‐anatomical mapping and fluoroscopy guidance.

All procedures were performed under sinus rhythm; internal (3–35 J) or external (50–220 J) electrical cardioversion was performed with gradually increasing shock intensity to restore sinus rhythm when AF was observed before or during the procedure.

Successful PVI was defined as bidirectional conduction block between the outside and the inside of the circumferential PVI area, confirmed by constant pacing from both sides and by electro‐anatomical mapping system using the circular mapping catheter. Conduction block from the PV to the LA was confirmed by high‐output pacing (20 V output, 1 ms pulse) from the isolated PV area.

### 2.3 AutMmark Analysis

RF power, generator impedance, and CF were continuously measured during each RF energy application. AID was also continuously measured using AID module in EnSite X, providing the two parameters: absolute AID (Ω) and %AID (= absolute AID/initial impedance, %). The distance between neighboring ablation points (inter‐lesion distance) was measured after each RF application.

### 2.4 Ex‐Vivo Experiment

We examined the correlation between AID and actual lesion size following RF using various power, duration, and CF settings. Freshly excised swine ventricle was mounted on a platform and placed in a thermoregulated bath filled with circulating normal saline maintained at 37°C to mimic intracardiac blood flow. A peristaltic pump generated laminar flow. RF energy was applied using TFSE from the epicardial surface in a unipolar mode between the catheter tip electrode and a grounding patch placed at the bottom of the bath. A calibrated roller pump (CoolPoint, Abbott) was connected to the catheter for delivering saline solution at 13 mL/min from the irrigation tip. The ablation electrode was manually positioned at perpendicular [90°], oblique [45°] and parallel [0°] contact angles to the tissue; RF energy was delivered at each angle. The RF ablation lesions were created using various RF durations (15–60 s), power (30–50 W) and CF (10–15 g). Absolute AID and %AID values were continuously measured using AID module in EnSite X. The myocardium was cross‐sectioned along the surface length at each lesion. A digital caliper was used for each measurement with a resolution of 0.1 mm of the lesion. The maximum depth (*d*), maximum diameter (*e*), depth at the maximum diameter (*c*), and surface maximum diameter (*a*) of the cross‐sectioned area were measured. Lesion volume was calculated in each ablation point using the following formulas (Supporting Information S1: Figure 1A): lesion volume = (1∕6) ×*π* × (*e* ^2^ × *d* + *c* × *a* ^2^ ∕ 2) [8, 9]. When a steam pop (SP) occurred, the RF application was stopped and was restarted at a different site. The volume of lesions with SPs was not measured and was excluded from the analysis.

### 2.5 AID‐Guided PVI

The clinical usability of AID was evaluated in another subset of AF patients. %AID‐guided PVI was performed using modified HPSD (RF power: ≥ 40 W, RF time: 15–20 s, contact force [CF]: 5–20 g). RF application was discontinued when the %AID reached a cutoff value for predicting CG or when RF energy was delivered for maximum duration (20 s). The RF application was discontinued when there was a spike in esophageal temperature (≥ 41°C) and when %AID (exceeded a threshold predicting SPs) to avoid adverse events. The use of anti‐arrhythmic drugs was allowed after the procedure but discouraged after the blanking period. The primary end point was successful first‐pass PVI. The secondary end points were recurrence of any atrial tachy‐arrhythmia (lasting 30 s) after the 3‐month blanking period and composite of adverse events including cardiac tamponade, esophageal fistula, phrenic nerve palsy, thromboembolism, heart failure hospitalization, cardiovascular death, and all‐cause death, during 1‐year follow‐up.

### 2.6 Statistical Analysis

Continuous variables, represented as mean ± standard deviation, were compared using unpaired *t*‐tests. Categorical data, expressed as frequencies and percentages, were compared using chi‐square tests. AF‐free survival rate was calculated using Kaplan–Meier survival analysis. Simple linear regression analysis was performed to examine the relationships between AID and lesion characteristics. Receiver operating curve (ROC) and area under the curve (AUC) were analyzed to determine the best cutoff of AID for predicting CGs and SPs; AUCs were statistically compared using DeLong′s test.

All tests were two‐sided, and a *p*‐value < 0.05 was considered statistically significant. Statistical analyses were performed using JMP17 (SAS Institute, Cary, NC, USA).

## 3 Results

### 3.1 First Study

Baseline characteristics of 20 patients were demonstrated in Table 1. All patients had no scar/low voltage area of the left atrium in the 3D mapping before PVI. One thousand six hundred eighty‐seven RF ablation points (AutoMarks) from 20 patients were analyzed. After first‐pass ablation, CGs were observed in 45 points (CG[+]) but not in 1642 (CG[−]). The success rates of first‐pass isolation were 70% in LPV and 70% in RPV. CG[+] had significantly lower RF energy, unfiltered generator impedance drop, absolute AID, and %AID than CG[−]. There were no statistical differences in CF and inter‐lesion distance between CG[+] and CG[−]　(Table 2).

**Table 1:** Patient characteristics.

	AF patient (*n* = 20)
Age, y/o	66.8 ± 11.5
Male, *n* (%)	14 (70)
BMI, kg/m2	25.3±2.9
Type of AF, persistent, *n* (%)	11 (55)
CHA_2_DS_2_‐VASc score, points	2.8±2.0
CHF, *n* (%)	8 (40)
HT, *n* (%)	15 (75)
DM, *n* (%)	5 (25)
Stroke/TIA, *n* (%)	2 (10)
Vascular Disease, *n* (%)	2 (10)
Medications, *n* (%)	
ACEI/ARB, *n* (%)	9 (45)
β blocker, *n* (%)	14(70)
AAD, *n* (%)	1 (5)
Laboratory data	
NT‐proBNP, pg/mL	703±630
Cr, mg/dL	0.81±0.19
Echocardiography	
LVEF, %	58±6
LAD, mm	41.3±7.0
HPSD/SPLD, *n*	10/10

Abbreviations: AAD, anti‐arrhythmic drug; ACEI/ARB, angiotensin converting enzyme inhibitor/angiotensin receptor blocker; AF, atrial fibrillation; BMI, body mass index; CHF, congestive heart failure; Cr, creatinine; DM, diabetes mellitus; LVEF, left‐ventricular ejection fraction; HT, hypertension; HPSD, high power short duration ablation; LAD, left‐atrial diameter; SPLD, standard power long duration ablation; TIA, transient ischemic attack.

**Table 2:** Comparison of RF parameters between CG[−] and CG[+].

	Total (*n* = 1687)	CG[−] (*n* = 1682)	CG[+] (*n* = 45)	*p* value
RF power, W	43.1±4.9	43.0±5.0	45.7±3.5	0.0003
RF energy, J	693±172	696±172	600±163	0.0002
ILD, mm	5.3±3.7	5.3±3.7	5.2±3.3	0.8787
Duration, sec	16.5±5.2	16.6±5.3	13.4±4.5	< 0.0001
CF, g	11.4±5.4	11.3±5.4	12.6±4.5	0.2455
Baseline impedance, Ω	93.7±9.4	93.7±9.5	93.1±7.8	0.6873
Unfiltered generator impedance drop, Ω	13.0±7.8	13.0±7.9	10.3±2.5	0.0214
Absolute AID, Ω	9.4±3.7	9.4±3.7	7.0±2.3	< 0.0001
%AID, %	9.6±3.0	9.6±3.1	7.2±1.7	< 0.0001

Abbreviations: AID, averaged impedance drop; CG, conduction gap; CF, contact force; ILD, inter‐lesion distance; RF, radiofrequency.

**Figure 2:**
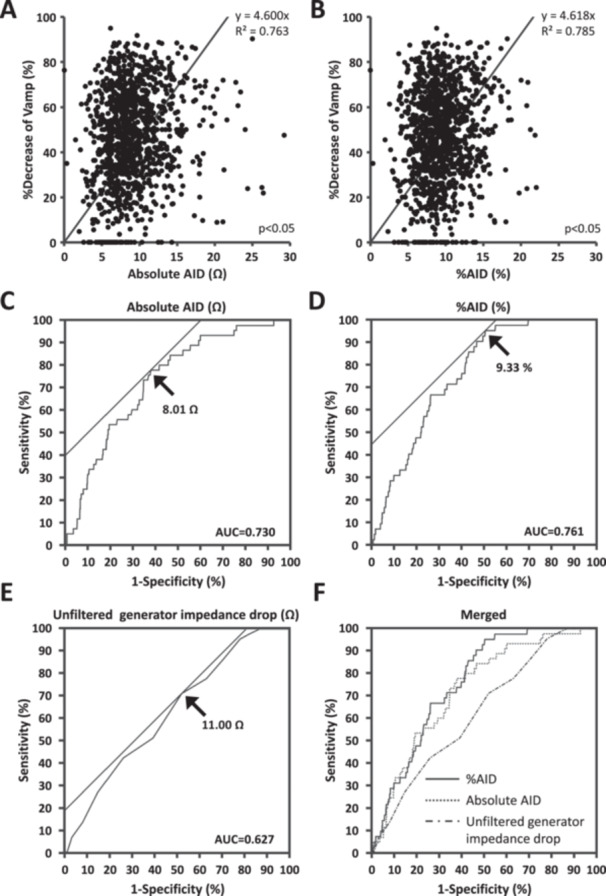
AID for predicting conduction gaps (CGs). (A) Correlation of %decrease of bipolar voltage amplitude (Vamp) with absolute AID. (B) Correlation of Vamp with %AID. (C) Cutoff of absolute AID for predicting CGs. (D) Cutoff of %AID for predicting CGs. (E) Cutoff of unfiltered generator impedance drop for predicting CGs. (F) Merged receiver operating characteristic curves.

Voltage amplitude (Vamp), recorded at the distal bipolar electrodes of TFSE, was analyzed before and after RF application. Absolute AID and %AID were linearly correlated with the percent decrease of Vamp (%Vamp = [Vamp before RF application – Vamp after RF application] × 100/Vamp before RF application, Figure 2A,B). The R‐squared values for absolute AID and %AID were 0.763 and 0.785, respectively.

In ROC curves analysis, the cutoff values of absolute AID, %AID, and unfiltered generator impedance drop for predicting CGs were 8.01 Ω, 9.33%, and 11.0 Ω, respectively (Figure 2C–E); AUC values for absolute AID and %AID were 0.730 and 0.761, respectively, and were significantly higher than the value in unfiltered generator impedance drop (AUC = 0.627, Figure 2F).

**Figure 3:**
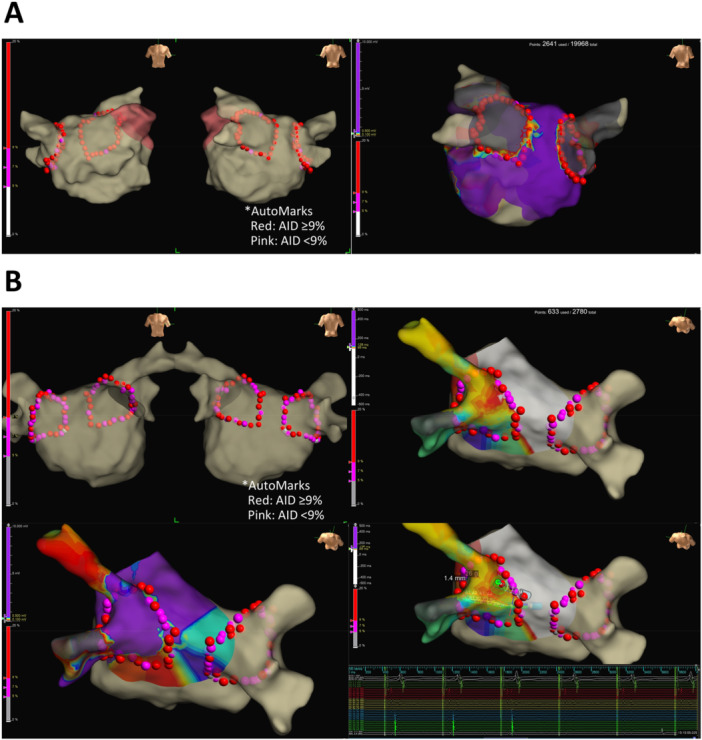
Representative cases with and without CGs. (A) A representative case without CGs. Red tags indicate RF lesions with %AID ≥ 9%. Pink tags indicate those with %AID < 9%. (B) A representative case with CGs.

Figure 3A shows a representative case of successful first‐pass PVI. Red tag indicates points in which %AID reached at least 9%; pink tag indicates those in which %AID did not reach 9%. In this case, %AID in almost all points reached 9% or more, resulting in successful first‐pass PVI. Figure 3B shows an example of unsuccessful first‐pass PVI. In this case, %AID in many lesions did not reach 9% and first‐pass ablation failed to complete PVI. CGs were observed in the points with %AID < 9% as shown by pink tags. PVI was completed by additional RF application in the areas with the CGs.

### 3.2 Supplemental Ex‐Vivo Study

Ex‐vivo experiments using swine hearts was supplementally performed to confirm AID and lesion volume. The lesion volume data (*n* = 444) were correlated with absolute AID and %AID (Supporting Information S1: Figures 1B,C). The R‐squared values for absolute AID and %AID were 0.703 and 0.711, respectively. SPs were observed in 14 points. The lesions with SPs had significantly higher absolute AID and %AID than those without (Supporting Information S1: Figure 1D,E). In ROC curve analysis, the cutoff values for predicting SPs were 17.4 Ω and 13.7% of absolute AID and %AID, respectively (Supporting Information S1: Figure 1F,G): AUC values for absolute AID and %AID were 0.884 and 0.907, respectively.

### 3.3 Second Study

**Figure 4:**
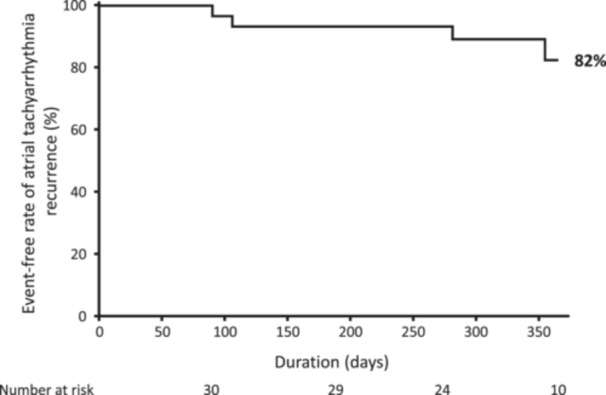
One‐year event‐free rate of atrial tachyarrhythmia recurrence.

We assumed that %AID is more useful than absolute AID in clinical practice since baseline impedance varies depending on patients; %AID is normalized by baseline impedance. In the second study, %AID was used as a parameter to estimate CGs. The protocol of HPSD was slightly modified to reduce CG (RF power: ≥ 40 W, RF time: 15–20 s, contact force [CF]: 5–20 g) based on the first protocol. The efficacy of %AID‐guided PVI was examined in another subset of AF patients (*n* = 30, Table 3); target %AID was set to ≥ 9% based on the first study. To avoid collateral damage and SPs, the RF application was stopped when esophageal temperature spiked (≥ 41°C) and/or when %AID suddenly plunged (> 14% based on the ex‐vivo study). The mean value of %AID was 10.5% ± 3.1% for LPV and 10.2% ± 3.2% for RPV in all PVI procedures. The success rate of first‐pass isolation was 90% in LPV and 90% in RPV. Compared to PVI with conventional HPSD/SPLD protocols in the first study, %AID‐guided PVI in the second study tended to improve the first‐pass isolation rate in the both LPV (90% vs. 70%, *p* = 0.074) and RPV (90% vs. 70%, *p* = 0.074) although it did not reach statistical significance. Table 4 shows analysis of RF parameters in all lesions (*n* = 2452 points): CGs were observed in 20 RF lesions but not in 2432. Unfiltered generator impedance drops, absolute AID, and %AID in CG[+] were significantly lower than the values in CG[−]; %AID showed the minimum p‐value among them (Table 4). SPs were not observed in all RF points. During the follow‐up, atrial tachyarrhythmias recurred in four patients; the event‐free rate of atrial tachyarrhythmia recurrence was 82% (Figure 4). No adverse events were observed at the procedure and during the follow‐up in all patients.

**Table 3:** Patient demographics and procedure characteristics in %AID‐guided ablation.

	AF patient (*n* = 30)
Age, y/o	69 ± 10
Male, *n* (%)	18 (60%)
BMI, kg/m2	24.5±4.6
Type of AF, persistent, *n* (%)	16 (67)
CHADS_2_ score, points	1.9±1.3
CHA_2_DS_2_‐VASc score, points	3.2±1.8
CHF, *n* (%)	17 (56%)
HT, *n* (%)	18 (60%)
DM, *n* (%)	7 (23%)
Stroke/TIA, *n* (%)	3 (10%)
Vascular Disease, *n* (%)	4 (13%)
Medications, *n* (%)	
ACEI/ARB, *n* (%)	15 (50%)
β blocker, *n* (%)	15 (50%)
AAD, *n* (%)	3 (10%)
Laboratory data	
NT‐proBNP, pg/mL	950±817
Cr, mg/dL	0.84±0.42
Echocardiography	
LVEF, %	55±8
LAD, mm	43.2±6.2
Successful first pass PVI	
LPV, *n* (%)	27 (90%)
RPV, *n* (%)	27 (90%)

*Note:* Same abbreviations as in Table 1.

**Table 4:** Comparison of RF parameters between CG[‐] and CG[+] in %AID‐guided ablation.

	CG [−] (*n* = 2432)	CG [+] (*n*=20)	*p*‐value
RF power, W	49 ± 4	49±1	0.347
RF energy, J	686±150	699±98	0.697
RF duration, sec	14.6±3.1	14.7±2.0	0.897
CF, g	12.1±5.6	9.7±3.4	0.061
RF power, W	49±4	49±1	0.347
Baseline impedance, Ω	95±9	93±11	0.364
Generator impedance drop, Ω	12.5±4.1	10.0±3.0	0.007
Absolute AID, Ω	10.1±3.6	7.7±2.5	0.003
%AID, %	10.4±3.2	8.1±1.8	0.001

*Note:* Same abbreviations as in Table 2.

## 4 Discussion

The major findings of this study are as follows. (1) The lesions with CGs had significantly lower AID than those without CGs. (2) AID was correlated with %Vamp. (3) The cutoff of %AID for predicting CGs was 9.33%. (4) In %AID (≥ 9%)‐guided PVI, the success rate of first‐pass isolation was 90% in LPV and 90% in RPV; the 1‐year AF‐free survival rate was 82%. To our best knowledge, this study is the first to demonstrate the efficacy of AID for estimation of RF lesion size and to show the efficacy of %AID‐guided PVI using TFSE.

### 4.1 Comparison With Previous Studies

Yamaguchi et al. also reported that the TFSE reduced the risk of SPs compared to TactiCath Ablation Catheter (TC, Abbott, St. Paul, MN, USA) in experimental model [8]. The percent decrease of generator impedance drop (%generator impedance drop) exhibits better predictability of SPs than the absolute value of generator impedance drop, which is similar to the results in this study.

Nair et al. evaluated the safety and effectiveness of TFSE for the treatment of drug‐refractory and symptomatic PAF [4]. The TFSE′s flexible tip improved its stability compared to the solid tip of previous catheters, possibly contributing to improving the efficiency of the procedures. PVI was performed using high power (40–50 W) and low power ablation (< 40 W), a similar protocol to our study (HPSD and SPLD). They demonstrated that TFSE is a safe and effective treatment of drug‐refractory PAF and enables more efficient procedures than previous‐generation catheters. However, this study has not examined the details of input/output parameters in each RF point and lesion characteristics.

Arai et al. compared the acute procedural safety among the latest four ablation catheters, THERMOCOOL SMARTTOUCH Surround Flow (STSF, Biosense Webster), QDOT Micro (QDM, Biosense Webster), TC, and TFSE cahteters [10]. The incidence of cardiac tamponade was significantly higher in the TC/TFSE group than the STSF/QDM group. Moreover, in the TC/TFSE group, the events of cardiac tamponade were increased by replacing TC with TFSE. One possible explanation why TFSE increased cardiac tamponade is that STSF/QDM and TC have lesion‐estimating parameters (Ablation Index for QDM/STSF and Lesion Square Index for TC) but TF does not. In the initial experiences, RF ablation using TFSE without any lesion‐estimating parameter might deliver too much energy to cause excessive heating, causing SPs and collateral damage.

Dello Russo A, et al. evaluated the clinical efficacy and safety of HPSD (40–50 W for 10–20 s)‐based PVI with the TFSE as compared to SPLD (30–35 W, targeting a Lesion Size Index of 4.0–5.5)‐based PVI with the TC [5]. They demonstrated that the HPSD group had significantly shorter procedure time than the SPLD group. The HPSD group also had higher success rate of first‐pass isolation than the SPLD group although the difference was not statistically significant (*p* = 0.063). The average of generator impedance drops in the HPSD group was significantly larger than the value in the SPLD group, implicating the larger lesion size in the HPSD ablation despite the shorter procedure time. There is no index to estimate lesion formation for TFSE; the clinical performance of TFSE would improve when a lesion‐estimating parameter becomes available for TFSE.

Matsumoto et al. performed PVI using TFSE with HPSD (Power: 50 W, CF: 5–20 g, Duration; 15–20 s); the success rates of the first‐pass isolation were 87% for LPV and 82% for RPV [11]. The cutoff values of the generator impedance drop to predict first‐pass isolation were 13.5 Ω for LPV and 14.5 Ω for RPV; the AUC was 0.612 for LPV and 0.586 for RPV. In this study, the best cutoff of %AID to predict CGs was 9.33% and the AUC was 0.761. In this study, the success rate of first‐pass isolation rates was 90% for LPV and 90% for RPV, which appears to be slightly higher than the values in the previous study. AID may improve the usability of impedance drop as an end point for RF application using TFSE. A previous study demonstrated that the first‐pass isolation was independent predictor for 1‐year AF recurrence after catheter ablation [12]. The higher first‐pass isolation in this study may contribute to the higher achievement of durable PVI.

In the first study, there were some lesions with CGs where %AID reached 9% (7/45 points); they were located in the anterior (ridge)/roof region of the LPV and the posterior region of the RPV antra. The anterior (ridge)/roof region of the LPV antrum has a thicker atrial wall than the other regions. The surface of posterior wall in the RPV antrum is sometimes curved due to compression by the vertebrae, which may cause the instability of RF catheter. Therefore, %AID ≥ 9% would be insufficient to guarantee durable lesions across all anatomical locations within the pulmonary vein antra. In contrast, there were also many lesions without CGs where %AID did not reach 9% in the first study (756/1642 points). This means that %AID ≥ 9% would not be necessary for all lesions to avoid CGs.

The variability of anatomy, wall thickness, and fibrotic remodeling especially at the PV antrum would have a significant impact on the lesion formation. The clinical environment and numerous factors beyond the biophysics of radiofrequency energy delivery (such as catheter stability, tissue contact angle, and convective cooling from blood flow) also substantially affect lesion formation. Tailoring %AID based on anatomical characteristics and clinical environment may increase the efficacy and safety of RFCA using TFSE.

We performed %AID‐guided PVI, and accomplished successful first‐pass isolation in most patients. Nevertheless, we failed first‐pass PVI in several patients; CGs were observed at areas showing low %AID (< 9%). RF application was discontinued due to the maximum duration of RF delivery (20 s) or the excessive rise of esophageal temperature before %AID reaches 9%. In these areas, our modified HPSD ablation might not deliver enough RF energy to create transmural lesion. The additional lesion modification would improve the acute success rate of PVI when %AID does not reach 9% (e.g. increasing CF, changing force direction, longer duration ablation using standard power).

SPs may cause cardiac tamponade. A previous study reported that the magnitude of impedance drop could predict SPs. Limiting RF energy to avoid massive impedance decrease (< 18 Ω) would reduce the likelihood of a SP [13]. However, the thresholds of impedance drop to predict a SP would differ between RF catheters; the information on TFSE is limited. In this study, the cutoff value of %AID was 14% for predicting SPs in ex‐vivo experiment; RF application was stopped when there was a spike in %AID ( > 14%) and no SPs were observed in the second protocol. When the baseline impedance was low, the amount of current through the tissue increased with the same output energy, resulting in a larger lesion size and a higher risk of SPs. Since being calculated as dividing absolute AID by baseline impedance, %AID would minimize the impact of the baseline impedance on risk prediction of SPs.

## 5 Limitations

This is a single‐center study with a small number of patients. The second study was a single‐arm study evaluating the %AID‐guided strategy; a lack of control group is a significant drawback in this study. We evaluated the acute success of first‐pass isolation in %AID‐guided PVI and assessed the outcomes of the PVI during 1‐year follow‐up. However, the long‐term durability of PVI for more than 1 year has not been examined. The %AID‐guided ablation targeting 9% yielded the higher success rate of first‐pass PVI without any SP. However, wall thickness varies among the regions surrounding PV. RF application tailoring AID based on the wall thickness in each region would be more effective and safer than the ablation using fixed cutoff of %AID. This study evaluated AF recurrence using 1‐week Holter monitoring but the results may differ when 2‐week Holter monitors or implantable loop‐recorders are used.

## 6 Conclusion

AID would improve the usability of impedance drop as an end point for RF application. %AID‐guided PVI using TFSE would be effective and safe in patients with AF.

## Funding

The authors received no specific funding for this work.

## Conflicts of Interest

M.H. received speaker fees from Abbott and Medtronic. Y.M., Y.N., A.N., E.W., Y.O., H.I. have nothing to disclose.

## Supporting information

Supplemental Figure Legends 20251005.

Supplemental Figure Legends 20251005.

## Data Availability

The deidentified participant data will be shared upon reasonable request. Please directly contact the corresponding author to request data sharing.
