# Supplementary material for: Averaged Impedance Drop Estimates Conduction Gap During Pulmonary Vein Isolation in Atrial Fibrillation
Source: J Cardiovasc Electrophysiol. 2026 Jan 9;37(3):501–9. doi: 10.1111/jce.70246 (PMC12980475; doi:10.1111/jce.70246)
Supplement: Supplementary file 1 — Supplemental Figure Legends 20251005. [file JCE-37-501-s002.docx]

**Supplemental Figure Legend.**

**Supplemental Figure 1. Ex-vivo experiments using swine hearts.** (A) RF application using TFSE and measurement of the RF lesion (left). Parameters for lesion volume calculation in cross-sectioned myocardium (right). (B) Correlation of absolute AID with lesion volume. (C) Correlation of %AID with lesion volume. (D) Absolute AID values at RF lesions with and without SPs. (E) %AID values at RF lesions with and without steam pops (SPs). (F) Cutoff of absolute AID for predicting SPs. (G) Cutoff of %AID for predicting SPs.
