# Supplementary material for: Averaged Impedance Drop Estimates Conduction Gap During Pulmonary Vein Isolation in Atrial Fibrillation
Source: J Cardiovasc Electrophysiol. 2026 Jan 9;37(3):501–9. doi: 10.1111/jce.70246 (PMC12980475; doi:10.1111/jce.70246)
Supplement: Supplementary file 2 — Supplemental Figure Legends 20251005. [file JCE-37-501-s001.pptx]

## Slide 1
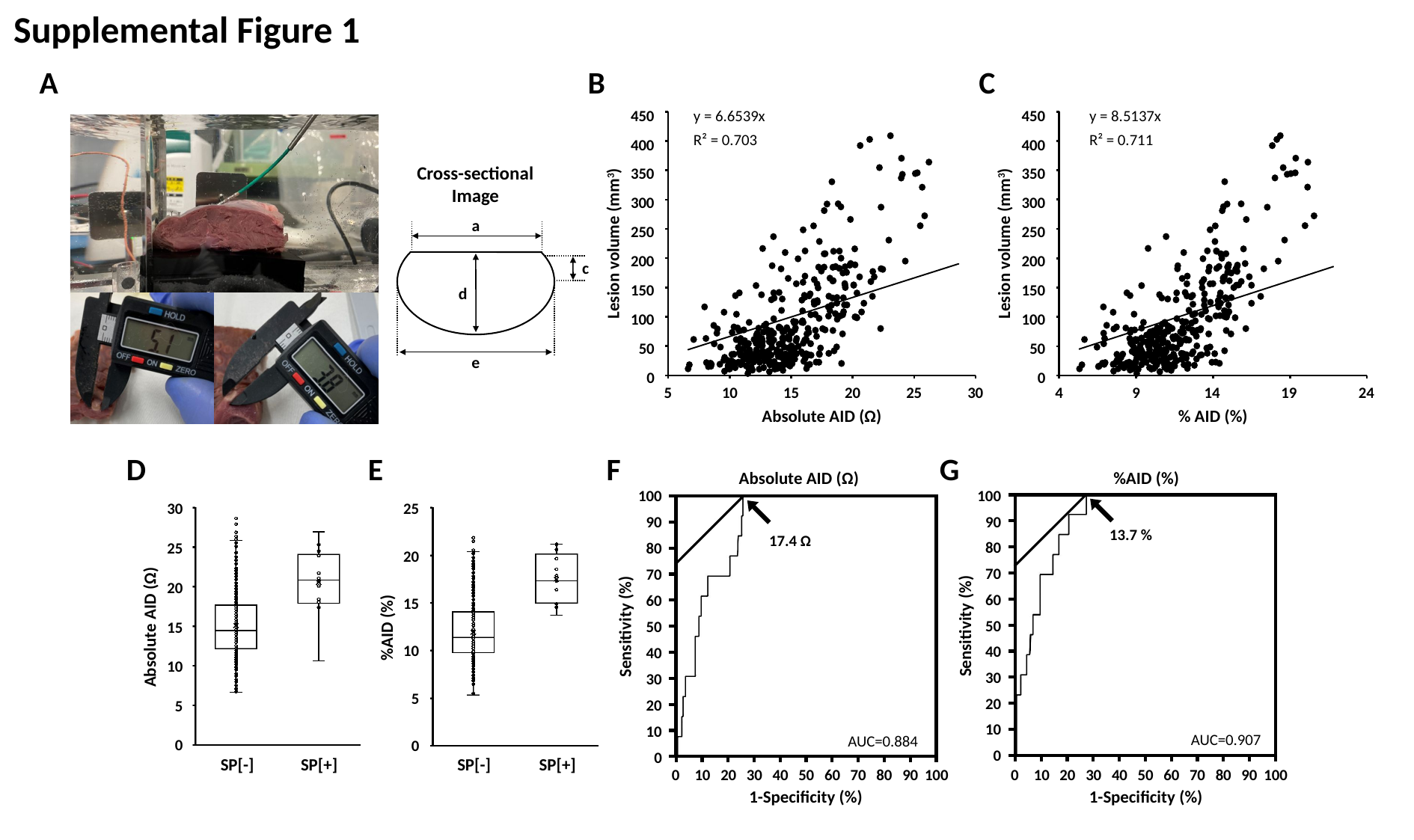

Supplemental Figure 1
A
B
C
450
y = 6.6539x
R² = 0.703
400
350
300
250
Lesion volume (mm3)
200
150
100
50
0
5
10
15
20
25
30
Absolute AID (Ω)
450
y = 8.5137x
R² = 0.711
400
350
300
250
Lesion volume (mm3)
200
150
100
50
0
4
9
14
19
24
% AID (%)
Cross-sectional Image
a
c
d
e
D
E
F
G
Absolute AID (Ω)
100
90
17.4 Ω
80
70
60
50
40
30
20
10
AUC=0.884
0
0
10
20
30
40
50
60
70
80
90
100
Sensitivity (%)
1-Specificity (%)
%AID (%)
100
90
13.7 %
80
70
60
50
40
30
20
10
AUC=0.907
0
0
10
20
30
40
50
60
70
80
90
100
Sensitivity (%)
1-Specificity (%)
30
25
20
15
10
5
0
Absolute AID (Ω)
SP[-]
SP[+]
25
20
15
10
5
0
%AID (%)
SP[-]
SP[+]
